# First Full-Scale 2D Field Experiment on Semi-Embedded Rubber Column Metamaterials: Enhanced Attenuation of Love Waves and Mechanistic Insights

**DOI:** 10.3390/ma18245517

**Published:** 2025-12-08

**Authors:** Xinchao Zhang, Ning Zheng, Changyin Ji, Yulin Lu, Qingfan Shi

**Affiliations:** 1 School of Physics, Beijing Institute of Technology, Beijing 100081, China; 2 Key Laboratory of Building Collapse Mechanism and Disaster Prevention, China Earthquake Administration, Institute of Disaster Prevention, Langfang 065201, China; 3 Hebei Technology Innovation Center for Multi-Hazard Resilience and Emergency Handling of Engineering Structures, Institute of Disaster Prevention, Langfang 065201, China

**Keywords:** seismic metamaterials, field experiments, Rayleigh wave, Love wave, seismic wave attenuation, local resonance

## Abstract

**Highlights:**

**What are the main findings?**

- This study conducts the first full-scale 2D field experiment to validate semi-embedded rubber column metamaterials (SEM) for seismic wave attenuation, revealing a global bandgap of 25–37 Hz and a localized bandgap of 37–42 Hz; at the central frequency of 31 Hz, SEM achieves −9.3 dB attenuation for Love waves and −5.3 dB for Rayleigh waves, with a notably more pronounced mitigation effect on Love waves.
- Theoretical and experimental analyses identify SEM’s unique energy dissipation mechanism: the exposed above-ground sections of rubber resonators dominate energy absorption via resonant oscillations, while the buried underground segments intro-duce damping that partially diminishes surface wave attenuation; additionally, low-er damping is correlated with enhanced surface wave attenuation.
- This research fills critical gaps: it addresses the lack of understanding regarding SEM’s real-world performance in mitigating different surface seismic waves, and specifically compensates for the long-standing shortage of field data on Love wave mitigation.

**What are the implications of the main findings?**

- For engineering applications, the findings provide valuable references for the practi-cal use of SEM in seismic protection systems, and offer actionable guidelines for flexi-ble resonator design—prioritizing damping characteristics to enhance attenuation efficiency, a point unreported in previous simulation or laboratory-based SEM stud-ies.
- For academic research, this pioneering full-scale 2D on-site validation bridges the gap between simulation-based predictions and practical seismic protection systems, lay-ing a foundation for future related studies on seismic metamaterials.
- For infrastructure protection, the results support the development of deployable and cost-effective seismic shields for vulnerable infrastructure (e.g., bridges, skyscrap-ers)—structures highly at risk from Love waves—strengthening their resilience against destructive seismic surface waves.

**Abstract:**

Despite recent numerical simulations and limited laboratory studies highlighting the potential of semi-embedded seismic metamaterials (SEM) in attenuating Rayleigh waves, their real-world effectiveness remains unverified, particularly for Love waves. Love waves pose significant destructive risks to slender structures but have rarely been the focus of research. To address this gap, we present the first full-scale 2D field experiment on an SEM composed of an array of semi-embedded rubber column resonators. The experimental results reveal a global bandgap spanning 25–37 Hz and a localized bandgap at 37–42 Hz. At the central frequency of the global bandgap (*f*_0_ = 31 Hz), the attenuation reaches −9.3 dB for Love waves and −5.3 dB for Rayleigh waves, with the mitigation of Love waves being notably pronounced. Furthermore, our theoretical and experimental analyses provide novel mechanistic insights: the primary energy dissipation in flexible rubber resonators arises from the resonance of their exposed above-ground sections, while the underground buried parts introduce damping that moderately reduces the efficiency of surface wave attenuation. This pioneering full-scale on-site validation bridges the critical gap between simulation-based predictions and practical seismic protection systems, providing valuable reference for the engineering application of SEM, especially for mitigating destructive waves.

## 1. Introduction

Recently, semi-embedded metamaterials (SEM) have garnered significant research interest due to their demonstrated ability to effectively attenuate Rayleigh surface waves, as evidenced by numerical simulations and laboratory experiments [1,2,3,4,5]. As a structural variant of locally resonant seismic metamaterials (RSM), SEM integrates the geometric and functional features of two classic RSM configurations—Buried RSM and Above-Surface RSM—combining the land-use efficiency of the former and the reduced subsurface disruption of the latter. This interest in SEM builds upon a decade of advancements in RSM research, which have offered a promising alternative to traditional seismic protection strategies by virtue of their sub-wavelength scale structures, enabling precise control of long-wavelength seismic vibrations [6].

Buried RSM include cylindrical tubes with suspended heavy cylinders [7], foam-wrapped steel columns [8], inertial resonators with rubber-ligament-connected iron spheres [9], and various pile/column designs [10,11,12,13,14,15,16,17,18,19]. Among these, periodic pile systems have been extensively studied through both laboratory and field experiments. For instance, Ma et al. [10] explored the attenuation zone of soil-periodic pile barriers, demonstrating their effectiveness in mitigating surface waves through controlled laboratory tests. Furthermore, Yan et al. [11] investigated seismic isolation using two-dimensional periodic foundations, examining their practical application in vibration mitigation. Another notable work by Wang et al. [12] focused on active isolation tests employing metamaterial-based barriers and foundations, revealing the significant role of periodic designs in enhancing vibration control efficacy. Beyond pile systems, experimental explorations by Shi et al. [13] using metaconcrete barriers as foundation pit backfill and by Nistri et al. [14] with modular metamaterial systems further highlight innovative approaches to buried resonator design, all validating promising attenuation performance. However, Buried RSM still face inherent challenges: soil nonlinearity and heterogeneity—exacerbated by variations in confining pressure, consolidation, and frequency—introduce uncertainty to their bandgap performance, while their installation may pose risks of geological disturbance and underground infrastructure disruption [15].

In contrast, Above-Surface RSM—encompassing mass–spring systems [16,17,18,19,20,21], rod-type structures [22,23,24,25], and fractal/pillar designs [17,26,27,28,29,30]—avoid subsurface disruption and have seen significant experimental advancements in recent years. Palermo et al. [16] validated Rayleigh wave attenuation using engineered metabarriers through large-scale laboratory tests, while their subsequent work [31] focused on Love wave control via resonant metasurfaces—though limited to small-scale models. Zaccherini et al. [25] conducted pioneering field experiments on granular media, confirming the attenuation of shear waves by locally resonant metasurfaces. Notably, Zeng et al. [29] demonstrated the effectiveness of inverted T-shaped seismic metamaterials in a scaled field experiment, achieving significant Rayleigh wave attenuation over a broad frequency range (34–130 Hz). Field-scale studies have even expanded to natural systems: Colombi et al. [32] identified Rayleigh wave bandgaps (30–45 Hz) in periodic forest arrays, demonstrating the potential of natural seismic metamaterials, while Lott et al. [33] further reinforced this by showing notable attenuation of Rayleigh waves within the 40–60 Hz range in forest arrays. Despite these strides, RSM research remains heavily reliant on numerical simulations and laboratory tests, with full-scale field experiments accounting for only a small fraction of studies [29,34,35,36,37,38,39,40]. Recent exceptions include Zhang et al. [19], who performed on-site validation of rubber oscillator arrays, and Halim et al. [35], who tested nested-mass metabarriers in real-world conditions—these works underscore the critical need for more extensive field verification to bridge the gap between theoretical predictions and practical applications.

While existing simulations and laboratory experiments have confirmed SEM’s efficacy in seismic wave attenuation [1,2,3,4,5], its translation to engineering applications demands further experimental validation under real-world conditions—a task complicated by technical challenges in field testing and the limitations of simplified theoretical models. As noted in [5], theoretical and numerical analyses frequently rely on idealized assumptions (e.g., homogenized soil models that overlook inherent soil heterogeneity or idealized boundary conditions), which inevitably create non-negligible discrepancies between simulations and real-world scenarios. For instance, a field study on partially embedded metamaterials [11] demonstrated their potential in attenuating Rayleigh waves by employing a composite unit of underground concrete platforms and aboveground steel–rubber structures, which leveraged resonant coupling between surface and subsurface components to reduce wave propagation. However, this and similar studies suffer from limited frequency coverage, primarily addressing Rayleigh waves within a narrow band (9–11 Hz), and fail to encompass broader frequency ranges or multiple wave types.

Critically, similar to other RSM research, SEM studies have thus far concentrated almost exclusively on Rayleigh waves, with minimal consideration paid to other destructive surface waves such as Love waves. This oversight is particularly significant for Love waves, which exhibit distinct propagation mechanics—their lateral shear forces pose a higher risk to slender or tall structures such as bridges and skyscrapers, thereby necessitating targeted attenuation strategies.

To bridge these research gaps, we conduct the first full-scale 2D field experiment to validate seismic wave attenuation by SEM, with a targeted focus on Love waves—a critical yet underexplored dimension in seismic metamaterial research. The design of the SEM is based on elastic wave propagation theory and numerical simulation techniques, featuring square rubber column resonators that are half-embedded underground and half-exposed above the surface. We measured vibration velocity signals in the x, y, and z directions using sensors placed behind the SEM, comparing scenarios with and without the SEM to assess their performance. The experimental results demonstrate that the SEM effectively attenuates seismic waves within the ultra-wideband range of 25–42 Hz, particularly for Love waves. By employing a simplified resonator model we developed and integrating field experimental results of flexible above-surface [19] and semi-embedded resonators, we further reveal the underlying physical mechanism: the primary energy dissipation in flexible rubber resonators arises from the resonance of their exposed above-ground sections, while the underground buried parts introduce damping that moderately reduces the efficiency of surface wave attenuation.

From an engineering perspective, this research provides actionable insights into seismic protection systems: the rubber-based SEM design offers cost-effectiveness and adjustable mechanical properties, facilitating practical implementation, and its ability to effectively attenuate Love waves can substantially lessen earthquake-induced damage to buildings and infrastructure. This pioneering full-scale on-site validation bridges the critical gap between simulation-based predictions and practical seismic protection systems, laying an experimental foundation for the future design and application of large-scale SEM-based protection systems in earthquake-prone areas.

## 2. Theory and Simulation

### 2.1. Mechanistic Analysis of Seismic Metamaterials

The propagation of elastic waves in a three-dimensional structure satisfies Lame’s equation

(1) $$\frac{E}{2(1-2\nu )(1+\nu )}\nabla (\nabla \cdot u)+\frac{E}{2(1+\nu )}\nabla^{2}u+\rho \omega^{2}u=0$$

where ***E*** is Young’s modulus, ***ν*** is Poisson’s ratio, ***ρ*** is medium density, ***u*** is the displacement field, and ***ω*** is the angular frequency of signal. The solution of an elastic wave propagating in a periodic potential field can be written as

(2) $$u(r,t)=u_{k}(r)\exp[i(k\cdot r-\omega t)]$$

where ***r*** is the space coordinate vector, ***k*** is the Bloch wave vector, and ***u_k_***(***r***) is a periodic function as follows:

(3) $$u_{k}(r+a)=u_{k}(r)$$

where ***a*** is the lattice vector. Accordingly, the Bloch periodic boundary conditions can be expressed as

(4) u(r+a,t)=u(r,t)exp[i(k⋅a)]

Substituting Equation (4) into Equation (1), we can obtain the eigenvalue equation

(5) $$[D(k)-\omega^{2}M]u=0$$

where ***D***(***k***) is the lattice stiffness matrix, ***M*** is the lattice mass matrix and ***k*** is the Bloch wave vector. By calculating, the wave vectors under different structures along the first Brillouin region M-Γ-X-M, the dispersion relationship between ***k*** and ω can be obtained.

### 2.2. Simulation of Semi-Embedded Seismic Metamaterials

**Figure 1:**
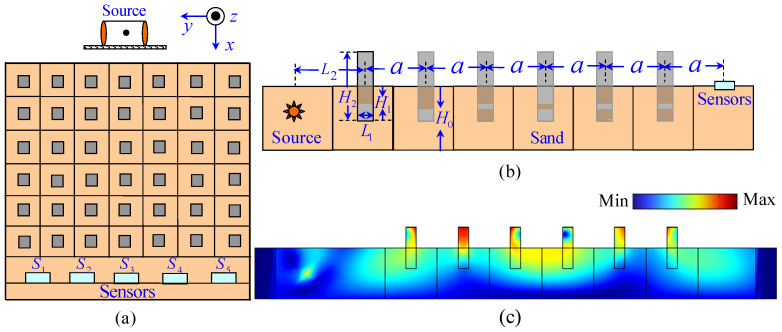
Schematic of the 2D SEM structure. (**a**) Top view; (**b**) Section view; (**c**) Wave propagation diagram.

Figure 1 illustrates the schematic diagrams of the SEM structure and the elastic wave propagation within the SEM. Subfigures a, b, and c, respectively, show the plan view, cross-sectional view, and wave propagation diagram. The structure comprises a 6 × 7 periodic array of semi-embedded square columns on a sand bed substrate with a depth of *H*_0_. A vibration source and five sets of sensors are symmetrically positioned on the front and rear sides of the periodic structure. The lattice constant of the structure is denoted as a, with each unit cell featuring a rectangular square column characterized by a side length *L*_1_, embedded depth *H*_1_, and total height *H*_2_. Table 1 and Table 2 give the parameters of the simulation.

**Figure 2:**
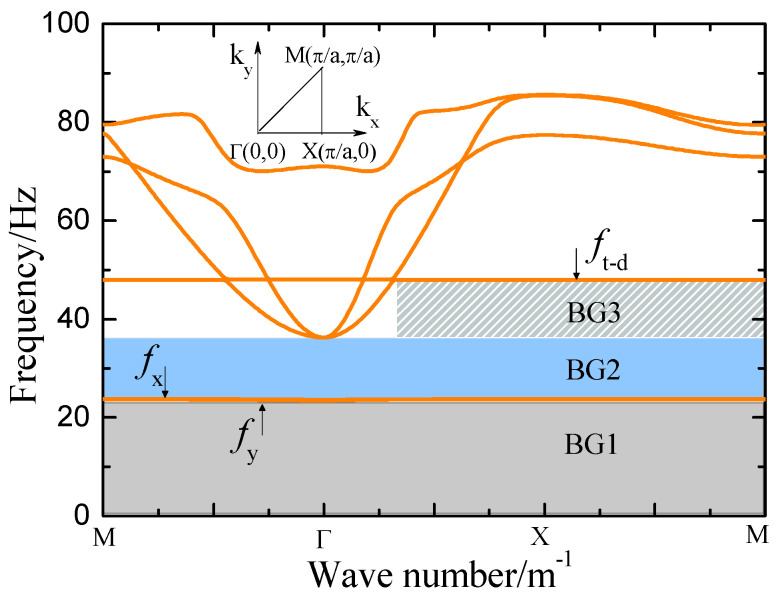
Band structure of the SEM.

Figure 2 shows the bandgap characteristics obtained from finite element simulations of the SEM using COMSOL Multiphysics, (5.2, COMSOL AB, Stockholm, Sweden) featuring two global bandgaps: 0–23 Hz (dark gray region, BG1) and 25–35 Hz (light blue region, BG2), along with a local bandgap spanning 36–49 Hz (light gray shaded region, BG3). To simulate wave propagation through the system, Equation (5) was employed in conjunction with Floquet–Bloch periodic boundary conditions applied to the unit cell. Due to the significantly lower Young’s modulus of the granular layer compared to the underlying soil, elastic waves undergo strong reflection at the sand–soil interface. Consequently, the bottom boundary of the substrate was set as a fixed constraint. The bandgaps BG1, BG2, and BG3 arise from the coupling of the fixed constraint with the vibration modes in the Y-direction, X-direction, and orsional vibration about the Z-axis, respectively. These findings provide critical insights for optimizing SEM in the design process.

**Figure 3:**
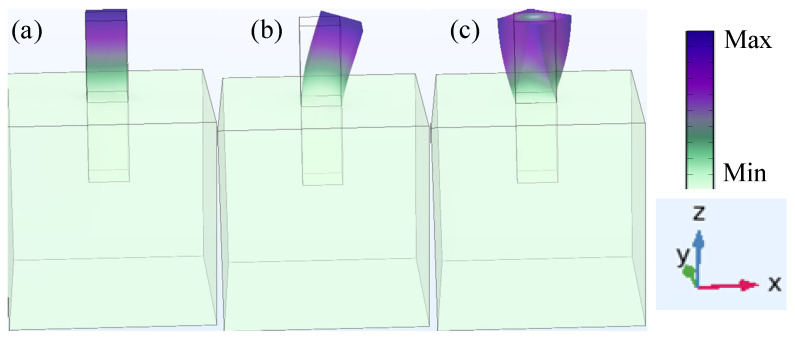
Vibration mode diagram of resonant unit. (**a**) *f*_y_ = 23.8 Hz; (**b**) *f*_x_ = 24.1 Hz; (**c**) *f_t-d_* = 49.2 Hz.

Figure 3 presents the vibration mode diagrams of the resonant unit simulated using COMSOL Multiphysics. Figure 3 a–c depict the vibration modes in the Y-direction, X-direction, and torsional mode about the Z-axis, respectively. The corresponding characteristic frequencies are 23.8 Hz, 24.1 Hz, and 49.2 Hz. Analysis of these modes indicates that the attenuation effect of the semi-embedded rubber resonator is primarily generated by its above-surface part.

### 2.3. Model of Resonators

**Figure 4:**
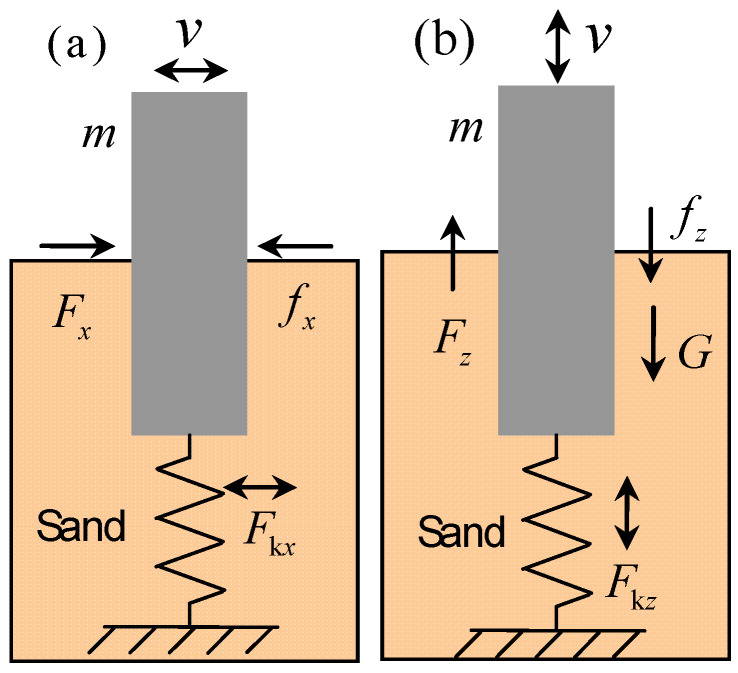
Schematic of surface resonator. (**a**) Horizontal force diagram; (**b**) Vertical force diagram.

Given that the mass–spring model serves as a widely accepted simplified representation for various types of resonators [16,17,18,19,20,21], it offers a convenient framework to explore different resonance mechanisms. To achieve this, we examine a single resonator to analyze the relationship between damping, natural frequency, and energy attenuation, as illustrated in Figure 4.

When the ground experiences forced vibrations, the driving force in the *x*-direction is $F_{x}=-m\overset{‥}{X}$, and the vertical forcing force is $F_{z}=-m\overset{‥}{Z}$, the elastic recovery force in the x-direction is $F_{kx}=-k_{1}x$ and the vertical elastic recovery force is $F_{kz}=-k_{2}z$, the air resistance in the x-direction is $f_{x}=-(b_{1}+b_{x})\dot{x}$ and the vertical air resistance is $f_{z}=-(b_{2}+b_{z})\dot{z}$. Here, m represents the mass of the vibrator, $k_{1}$ and $k_{2}$ are the stiffness coefficients of the spring in the horizontal and vertical directions, respectively. $b_{1}$ and $b_{x}$ are the air and sand resistance constants in the x-direction, while $b_{2}$ and $b_{z}$ represent the resistance constants in the vertical direction. *x* and *X* denote the displacements of the oscillator and ground vibration, respectively. Since the force analysis of the resonator in the y-direction is identical to that in *x*-direction, it is omitted here.

Assuming ground displacement caused by excitation is $X=A_{1}\cos\omega t$ and $Z=A_{2}\cos\omega t$, we can easily obtain the forced vibration equations of the oscillator along the horizontal and vertical directions [19]:

(6) $$\left\{\begin{array}{l} \overset{‥}{x}+2\beta_{x}\dot{x}+\omega_{x_{0}}^{2}x=A_{1}\omega^{2}\cos\omega t\\ \overset{‥}{z}+2\beta_{z}\dot{z}+\omega_{z_{0}}^{2}z=A_{2}\omega^{2}\cos\omega t+g \end{array}\right.$$

and their steady-state solutions are

(7) $$\left\{\begin{array}{l} x=A_{x}\cos(\omega t-\varphi_{x})\\ z=A_{z}\cos(\omega t-\varphi_{z})+g/\omega_{z_{0}}^{2} \end{array}\right.$$

where

(8) $$\left\{\begin{array}{l} A_{x}=\frac{A_{1}\omega^{2}}{\sqrt{{\left(\omega_{x_{0}}^{2}-\omega^{2}\right)}^{2}+4\beta_{x}^{2}\omega^{2}}}\\ A_{z}=\frac{A_{2}\omega^{2}}{\sqrt{{\left(\omega_{z_{0}}^{2}-\omega^{2}\right)}^{2}+4\beta_{z}^{2}\omega^{2}}} \end{array}\right.,\left\{\begin{array}{l} \varphi_{x}=\arctan\frac{2\beta_{x}\omega }{\omega_{x_{0}}^{2}-\omega^{2}}\\ \varphi_{z}=\arctan\frac{2\beta_{z}\omega }{\omega_{z_{0}}^{2}-\omega^{2}} \end{array}\right.$$

In Equation (8), $A_{x}$ and $A_{z}$ are the amplitudes, $\varphi_{x}$ and $\varphi_{z}$ are the initial phases, $\beta_{x}=\frac{b_{1}+b_{x}}{2m}$ and $\beta_{z}=\frac{b_{2}+b_{z}}{2m}$ are the damping coefficients, $\omega_{0x}^{2}=k_{1}/m$ and $\omega_{0z}^{2}=k_{2}/m$ are the natural frequencies for the *x*- and *z*-directions, respectively. Further, if we define the amplitude attenuation coefficient as

(9) $$\left\{\begin{array}{l} K_{x}=\frac{A_{x}}{A_{1}}=\frac{\omega^{2}}{\sqrt{{\left(\omega_{x_{0}}^{2}-\omega^{2}\right)}^{2}+4\beta_{x}^{2}\omega^{2}}}\\ K_{z}=\frac{A_{z}}{A_{2}}=\frac{\omega^{2}}{\sqrt{{\left(\omega_{z_{0}}^{2}-\omega^{2}\right)}^{2}+4\beta_{z}^{2}\omega^{2}}} \end{array}\right.$$

then $K_{x}$ and $K_{z}$ can be used to feedback the amplitude changes at different excitation frequencies. Equation (9) will be used to analyze the damping effect of the buried part of SEM on the attenuation of seismic waves.

## 3. Field Experimental Methodology

### 3.1. Set up

**Figure 5:**
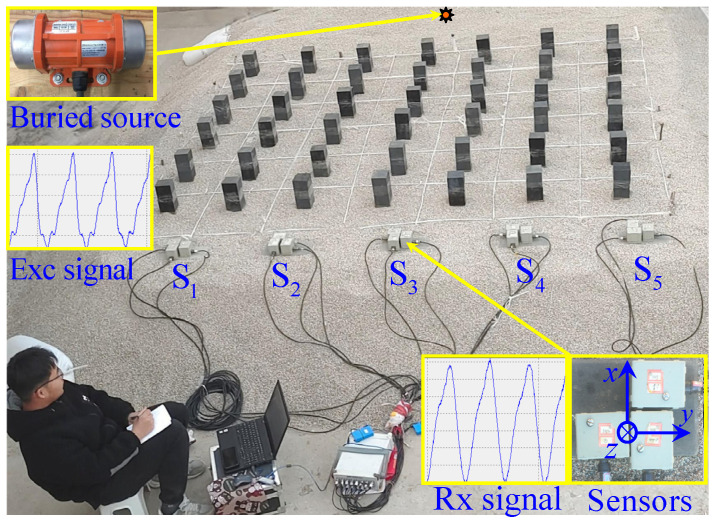
Photos of experimental site.

Figure 5 presents photographs of the experimental site, a set of sensors, and the vibration source. A granular layer was selected as the elastic wave propagation medium due to its uniform and idealized physical properties—these simplify theoretical analysis and parameter validation, a common approach in assessing the feasibility of seismic-resistant structures [35,41]. The granular base measures 5.0 × 4.0 × 0.5 m, using coarse sand particles with diameters ranging from 1 to 3 mm. In fact, the sand bed serves as a standardized wave propagation medium, analogous to acoustic water tanks in ultrasonics or uniform substrates in photonics. While it does not fully replicate the complexity of on-site stratified soils (e.g., clay content, bedding structure), it exhibits certain relevance to the physical properties of shallow sandy soils in earthquake-prone regions [42]. Rubber blocks were chosen as resonators for their excellent damping and energy absorption capabilities; their hardness and elastic properties are highly adjustable and controllable, facilitating processing and installation. According to simulation results, these rubber blocks (0.1 × 0.1 × 0.4 m) were arranged periodically as an oscillator array to form the SEM. The ambient temperature was between 5–10 °C, with site humidity at approximately 10%. The SER structure was constructed atop the granular layer based on simulation results. Five sets of sensors (signal type: vibration velocity, bandwidth: 0.5–150 Hz, low-frequency limit: −3 dB) and a 24-bit data acquisition system were used to collect the data. The sensors were placed 0.7 m apart, as depicted in Figure 1a, with their actual positions illustrated in Figure 5 and labeled S_1_ through S_5_. Each set consists of three unidirectional sensors to capture vibration signals in the x, y, and z directions (Figure 5).

The vibration source, powered by a 24 V DC battery, was an eccentric wheel vibrator (Model PT-ME6000CB/24-30, Shenzhen Putian Vibration Motor Co., Ltd., Shenzhen, China.) with a maximum excitation force of 0.7 kN (Figure 5). Its frequency is continuously adjustable within the range of 20–70 Hz, and it was embedded in the center of a large sand layer to generate vibrations. Due to minimal differences between excitation and background energy at lower frequencies, the lower limit of the experimental frequency range was set at 25 Hz, with data recorded at 3 Hz intervals up to 61 Hz. Data was considered valid when the dominant frequency recorded by the sensors matched the vibration frequency of the source. Vibration energy, calculated as the average energy per unit time through numerical integration, was regarded as effective when it exceeds five times the background energy. The experimental process involved first measuring the background energy, followed by measuring the energy of waves passing through the medium with and without SEM under different frequency excitations.

### 3.2. Data Processing

First, start the data acquisition instrument and wait for 5–10 s, then start the vibrator. During the experiment, record data for 120 s and calculate the average energy per unit time from the data collected between 40 s and 80 s, i.e.,

(10) $$\left\{\begin{array}{l} {\bar{E}}_{i}=\frac{1}{t_{2}-t_{1}}\int_{t_{1}}^{t_{2}}\frac{1}{2}mv_{i}^{2}dt\;\;\\ E=\sum_{i}E_{i} \end{array}\right.(i=x,y,z)$$

In which, ${\bar{E}}_{i}$ represents the components of energy in *x*, *y* and *z* directions, E is the total energy, *t*_2_ and *t*_1_ are the upper and lower limits of time, *v_i_* represents the corresponding velocity components, and m means the mass of sensor.

The energy with and without SEM is defined as ${\bar{E}}_{0}$ and ${\bar{E}}_{S}$, respectively. The corresponding components are defined as ${\bar{E}}_{0x}$, ${\bar{E}}_{0y}$, and ${\bar{E}}_{0z}$ for the former, and ${\bar{E}}_{Sx}$, ${\bar{E}}_{Sy}$ and ${\bar{E}}_{Sz}$ for the latter. The attenuation coefficients of total energy and partial energy for the SEM are defined as $K_{S}$ and $K_{Si}(i=x,y,z)$, respectively.

(11) $$\left\{\begin{array}{l} K_{S}=10\times \mathrm{lg}(\frac{{\bar{E}}_{S}}{{\bar{E}}_{0}})\\ K_{S_{i}}=10\times \mathrm{lg}(\frac{{\bar{E}}_{S_{i}}}{{\bar{E}}_{0i}}) \end{array}\right.$$

## 4. Field Experimental Results

### 4.1. Attenuation of Total Surface Wave Energy

**Figure 6:**
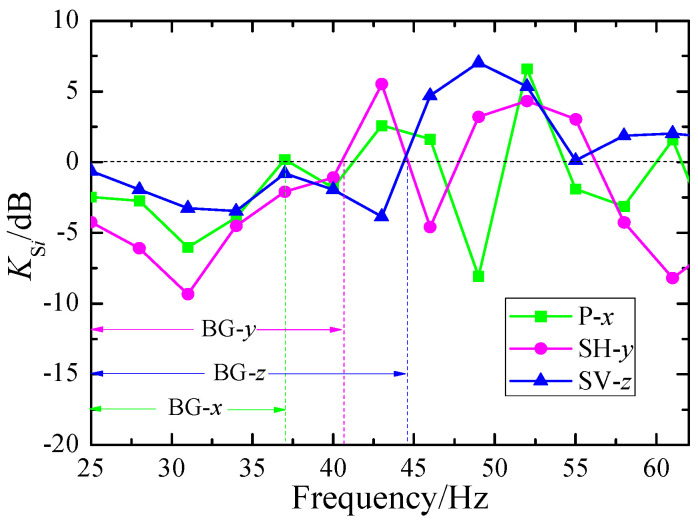
Energy attenuation of the vibration components at different frequencies.

We first investigated the attenuation of surface waves with different frequencies recorded by the sensor located at the central location S_3_ after they passed through the SEM, as illustrated in Figure 6.

Here, the green squares, pink circles and blue triangles correspond to the attenuation in the x-, y-, and z-directions, respectively. The bandgap ranges for waves in the x (P wave), y (SH wave), and z (SV wave) directions are identified as BG-x (25–37 Hz), BG-y (25–41 Hz), and BG-z (25–44 Hz). Obviously, the attenuation effect is more pronounced for horizontal waves compared to vertical waves.

**Figure 7:**
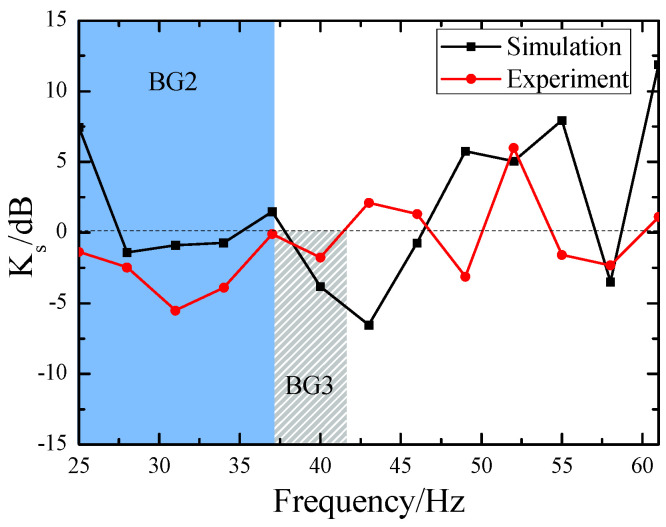
Attenuation diagram of total surface energy.

Based on the observational data depicted in Figure 6, we have obtained the attenuation data for the total energy of surface waves, as illustrated by the red dots in Figure 7. The bandgaps observed in the experimental results align closely with the global bandgap BG2 (25–37 Hz) and local bandgap BG3 (37–42 Hz) computed in Figure 3. Furthermore, we employed the commercial FEM-based simulation software, Comsol Multiphysics, to calculate the total energy attenuation of surface waves crossing the SEM, with the attenuation coefficients $K_{s}^{\prime }$ shown as the black square data points in Figure 7. The calculation was performed using Formula (12), which is highly analogous to Formula (11). In comparison with the experimental findings, the bandgap of BG2 is slightly narrower, ranging from 27 Hz to 35 Hz, whereas the bandgap of BG3 is slightly broader, spanning from 38 Hz to 46 Hz. Despite certain discrepancies between the experimental and simulation results, these findings are sufficient to demonstrate that the SEM structure is indeed effective in attenuating the energy of surface waves.

(12) $$K_{s}^{\prime }=10\times \mathrm{lg}\frac{E_{s}^{\prime }}{E_{0}^{\prime }}$$

### 4.2. Attenuation of Rayleigh and Love Wave Energy

Next, we analyze the attenuation effects of the SEM on surface waves based on the data recorded by the S_3_ sensor. In particular, Love waves are the primary focus of our study. Considering that Love waves are the result of the superposition of horizontally propagating shear waves (SH waves) and reflected waves at the granular layer interface, they should be included in the y-direction measurements. On the other hand, Rayleigh waves are a combination of horizontally propagating P waves and vertically polarized shear waves (SV waves), so they should be included in the x-direction and z-direction measurements. It is important to note that both Love waves and Rayleigh waves are the results of the interaction between the incident waves and the SEM. We, respectively, define the energy attenuation coefficients of Love waves and Rayleigh waves as follows.

(13) $$\left\{\begin{array}{l} K_{SL}=10\times \mathrm{lg}(\frac{{\bar{E}}_{Sy}}{{\bar{E}}_{0y}})\\ K_{SR}=10\times \mathrm{lg}(\frac{{\bar{E}}_{Sx}+{\bar{E}}_{Sz}}{{\bar{E}}_{0x}+{\bar{E}}_{0z}}) \end{array}\right.$$

**Figure 8:**
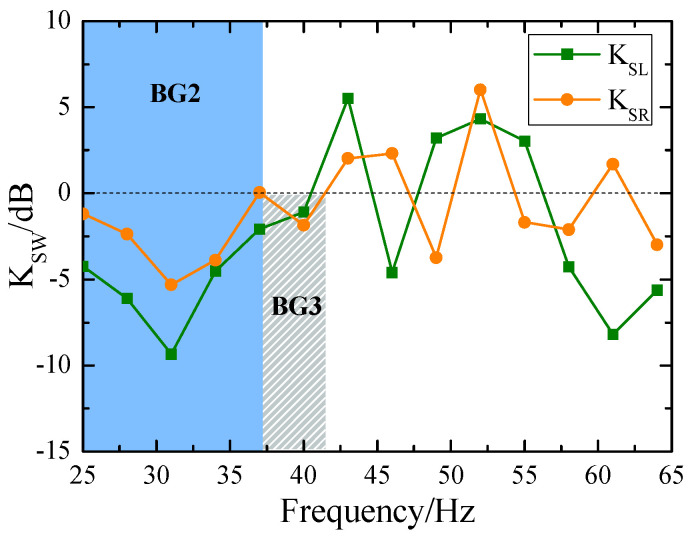
Attenuation of Rayleigh and Love waves at the central location S_3_.

Figure 8 illustrates the attenuation coefficients of Rayleigh and Love waves, calculated using Equation (13). In this figure, the orange circles denote the attenuation of Rayleigh waves, whereas the green squares represent the attenuation of Love waves. It is evident that the SEM is more effective at attenuating Love waves compared to Rayleigh waves. Within the frequency range of bandgaps, the attenuation reaches −9.3 dB for Love waves and −5.3 dB for Rayleigh waves at the center frequency (*f*_0_ = 31 Hz).

**Figure 9:**
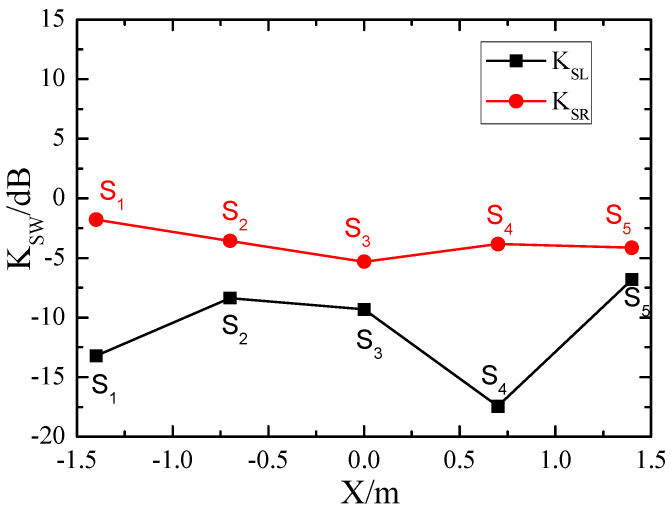
Attenuation of Rayleigh and Love waves at different locations.

In addition to the central location directly facing the wave source, we also examined the attenuation coefficients of Rayleigh waves (red circles) and Love waves (black squares) at other locations at the center frequency of *f*_0_ = 31 Hz, as illustrated in Figure 9. It can be observed that at all locations (S_1_–S_5_), the SEM attenuates Love waves more strongly than Rayleigh waves.

## 5. Discussion

Given that the SEM is partially above ground and partially underground, the corresponding contribution of each section to seismic surface wave attenuation clearly warrants investigation. It would be instructive to compare our current results with the in situ measurements obtained from our previous resonant metasurfaces (RMS) [19], especially since the above-ground structures of SEM and RMS′s structures (above-surface resonators) are completely identical.

**Figure 10:**
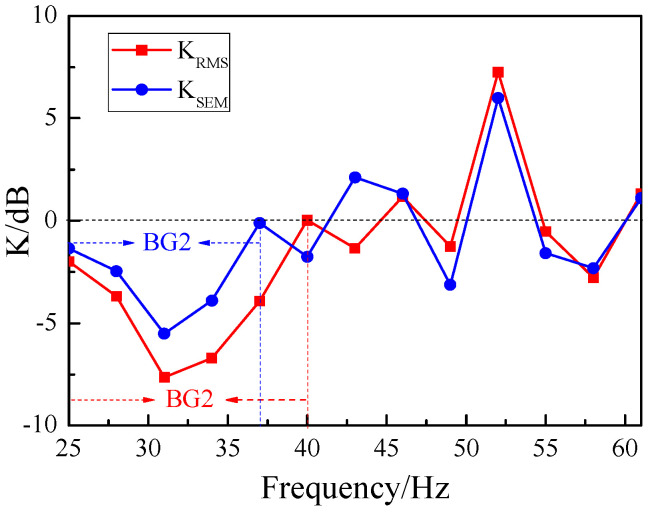
Attenuation of total energy: SEM vs. RMS.

Figure 10 presents a comparative attenuation plot of the total energy for the RMS and SEM at the central location (S_3_). In the figure, the blue circles represent the energy attenuation data for the SEM, while the red squares depict the energy attenuation data for the RMS. As can be observed from the plot, the RMS exhibits greater energy attenuation and a wider bandgap compared to the SEM. Specifically, at the central frequency *f*_0_ = 31 Hz, the energy attenuation of the RMS reaches −7.6 dB, whereas that of the SEM is −5.5 dB.

Considering that the vibration mode illustrated in Figure 2 has revealed that the energy attenuation of the flexible material primarily stems from the above-ground portion of the rubber resonator, it is logically reasonable to believe that the differences in energy attenuation between the SEM and RMS mainly originate from their subsurface structural differences. We know that the SEM has an additional buried-underground component in its structure compared to the RMS, so the damping of the SEM is relatively larger than that of the RMS. According to Formula (9), a larger damping coefficient β leads to a smaller energy attenuation coefficient K for surface waves, and thus a smaller energy attenuation for surface waves.

**Figure 11:**
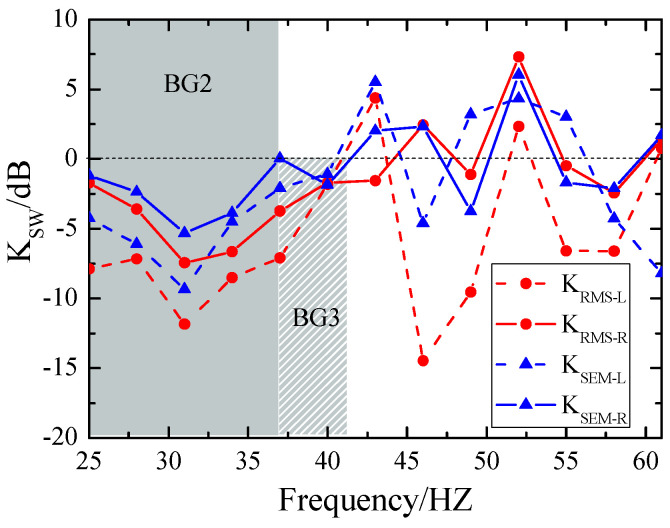
Attenuation of surface waves: SEM vs. RMS.

Next, we further examine the impact of damping on the energy attenuation of Rayleigh and Love waves. Since these waves propagate primarily along the Earth’s surface and constitute the major portion of surface seismic wave energy, damping inevitably influences their propagation. Figure 11 compares the energy attenuation curves of Rayleigh and Love waves for the RMS and SEM metastructures. For RMS, Rayleigh wave attenuation is represented by a solid line with red circles, while Love wave attenuation is denoted by a dashed line with red circles. For SEM, Rayleigh wave attenuation is shown as a solid line with blue triangles, and Love wave attenuation as a dashed line with blue triangles. It can be observed that RMS, with its lower damping, exhibits superior attenuation effects for both Love and Rayleigh waves compared to SEM. Based on these findings, we conclude that in seismic metamaterials dominated by local resonance modes, lower damping corresponds to more efficient surface wave attenuation. This discovery suggests that damping must be considered a key physical parameter when designing seismic metamaterials with flexible resonator array structures.

Building on this insight, we will consider computing the complex band structure as a future research direction [43] and focus on more complex viscoelastic models in our subsequent work [44] to better capture and quantify the damping effects highlighted in our findings.

## 6. Conclusions

This study presents the first full-scale 2D field experimental validation of semi-embedded rubber column metamaterials (SEM) for seismic wave attenuation, with a particular focus on Love waves—a critical yet underexplored surface wave type posing heightened risks to slender structures like bridges and skyscrapers. The experimental results demonstrate that SEM can effectively attenuate seismic waves, exhibiting a global bandgap spanning 25–37 Hz and a localized bandgap in the 37–42 Hz range. Notably, at the central frequency of 31 Hz, the attenuation reaches −9.3 dB for Love waves and −5.3 dB for Rayleigh waves, indicating a more pronounced effect on Love waves. This finding fills a critical gap in understanding the real-world performance of SEM in mitigating different types of surface seismic waves, especially addressing the long-standing lack of field data on Love wave mitigation.

Theoretical and experimental analyses reveal a key energy dissipation mechanism unique to SEM: the exposed above-ground sections of rubber resonators dominate energy absorption through resonant oscillations, while the damping effect of buried underground segments partially diminishes surface wave attenuation. This insight highlights a fundamental trade-off between resonator flexibility and damping-induced energy loss. Crucially, lower damping correlates with enhanced surface wave attenuation, underscoring the need to prioritize damping characteristics in flexible resonator design—an actionable guideline unreported in previous simulation or laboratory-based SEM studies.

In conclusion, this work successfully demonstrates the feasibility of SEM as seismic metamaterials for mitigating surface seismic waves. It bridges the gap between simulation-based predictions and real-world applications, providing practical guidelines for the development of deployable and cost-effective seismic shields in vulnerable infrastructure.

## Figures and Tables

**Table 1:** Geometric parameters.

*a* (m)	*L*_1_ (m)	*L*_2_ (m)	*H*_0_ (m)	*H*_1_ (m)	*H*_2_ (m)
0.5	0.1	1.0	0.5	0.2	0.4

**Table 2:** Material parameters.

Materials	Density ρ (kg/m^3^)	Young’s Modulus E (MPa)	Poisson’s Ratio ν
Sand	1400	20	0.35
Rubber	1500	7.8	0.47

## Data Availability

The raw data supporting the conclusions of this article will be made available by the authors on request.

## Abbreviations

The following abbreviations are used in this manuscript:

SEM	Semi-embedded rubber column metamaterials.
RSM	Locally resonant seismic metamaterials.
